# Population structure and genetic diversity of the giant anteater (*Myrmecophaga tridactyla*: Myrmecophagidae, Pilosa) in Brazil

**DOI:** 10.1590/1678-4685-GMB-2016-0104

**Published:** 2017-02-13

**Authors:** Camila L. Clozato, Flávia R. Miranda, Paula Lara-Ruiz, Rosane G. Collevatti, Fabrício R. Santos

**Affiliations:** 1 Laboratório de Biodiversidade e Evolução Molecular, Departamento de Biologia Geral, Universidade Federal de Minas Gerais (UFMG), Belo Horizonte, MG, Brazil.; 2 Projeto Tamanduá, São Paulo, SP, Brazil.; 3 Laboratorio de Genética e Biodiversidade, Instituto de Ciências Biológicas, Universidade Federal de Goiás (UFG), Goiânia, GO, Brazil.

**Keywords:** Giant Anteater, Xenarthra, Cerrado, genetic diversity, population structure

## Abstract

The giant anteater (*Myrmecophaga tridactyla*, Pilosa, Linnaeus 1758) belongs to the mammalian order Pilosa and presents a large distribution along South America, occupying a great variety of habitats. It is listed in the IUCN Red List of threatened species as Vulnerable. Despite threatened, there is a lack of studies regarding its genetic variability. The aim of this study was to examine the genetic diversity and patterns of genetic structure within remaining populations. We analyzed 77 individuals from seven different populations distributed in four biomes across Brazil: Cerrado, Pantanal, Atlantic Forest and Amazon Forest. We sequenced two mitochondrial markers (control region and Cyt-b) and two nuclear markers (AMELY and *RAG2*). We found high genetic diversity within subpopulations from National Parks of Serra da Canastra and Emas, both within the Cerrado biome, with signs of population expansion. Besides, we found a notable population structure between populations from the Cerrado/Pantanal and Amazon Forest biomes. This data is a major contribution to the knowledge of the evolutionary history of the species and to future management actions concerning its conservation.

## Introduction

The giant anteater, *Myrmecophaga tridactyla* Linnaeus 1758, is a mammal of the Myrmecophagidae family, order Pilosa (Gardner, 2005). It is the largest of all four anteater species and it occupies a great variety of habitats, such as rainforests, dry forests, wetlands and open fields (Fonseca and Aguiar, 2004). The species historical distribution corresponds to the area from Honduras in Central America to the Gran Chaco region of Bolivia, Paraguay and Argentina, and southern Pampas of Uruguay and Brazil in South America. The animals typically display solitary behavior and females give birth to a single young once a year after a 190 days of gestation (Eisenberg and Redford, 1999). They feed on ants and termites, and have a low metabolic rate and body temperature (McNab, 1985; Shaw *et al.*, 1985).

The giant anteater is the only member of its family listed in the World Conservation Union's 2014 IUCN Red List of Threatened Species as Vulnerable (VU) (Miranda *et al.*, 2014). The population numbers are declining along its range and the species is already extinct in some locations and countries, for instance, Uruguay, and possibly also Belize, Costa Rica and Guatemala (Fallabrino and Castiñeira, 2006; Miranda *et al.*, 2014). Moreover, within Brazil the species is critically threatened or even virtually extinct (not recorded for a long time, or rarely visualized) from a few states, such as Rio de Janeiro, Espírito Santo, Santa Catarina and Rio Grande do Sul (Bergallo *et al.*, 2000; Fontana *et al.*, 2003; Cherem *et al.*, 2004; Lorenzutti and Almeida, 2006) and appears listed as threatened in 19 Brazilian states according to the national red list of threatened species published by ICMBio (Miranda *et al.*, 2014).

The main causes for the observed population decline are the deterioration and reduction of natural habitats (Fonseca *et al.*, 1999), along with hunting for food, skin trade and pet purposes (Leeuwenberg, 1997; Peres, 2001; Ferreira *et al.*, 2013), frequent road kills (*e.g*. Cunha *et al.*, 2010; Diniz and Brito, 2013), and extensive wildfires in natural parks that usually kill hundreds of animals at once (Silveira *et al.*, 1999). In addition, the species' natural characteristics also contribute to increase its vulnerability, such as the solitary habit with a long gestation time (Eisenberg and Redford, 1999), and the low metabolic rate that contributes to their slow movement (McNab, 1985) and makes them more susceptible to anthropic hunting.

These elements altogether are expected to make the remaining *M. tridactyla* populations progressively more isolated. It is known that reductions in size and range of populations increase their vulnerability to stochastic extinction, leading in several instances to local extinction (Gilpin and Soulé, 1986). Currently, it is largely accepted that genetic variability plays an important role in the persistence and adaptation of populations to changing environments (Lande and Shannon, 1996; Frankham *et al.*, 2002), and the loss of adaptive genetic diversity places wild populations in greater extinction risk (Frankham, 2005). Thus, the knowledge of population diversity within remaining populations of *M. tridactyla* is essential to aid in conservation management.

Regarding the giant anteater, Collevatti *et al.* (2007) performed a population genetic study with individuals from the National Park of Emas, one of the largest populations of the species in Brazil (Miranda *et al.*, 2006), using five microsatellite loci previously described by Garcia *et al.* (2005). The study revealed a marked inbreeding within the population, associated to low levels of polymorphism in all loci. Apart from that, no other population genetics or phylogeography studies have focused on *M. tridactyla.* The lack of scientific information concerning the species' diversity pattern and its population structure calls for detailed research studies on this threatened species.

In this study we present results on population genetic diversity and structure based on the analysis of mtDNA and nDNA markers in different remaining populations across the distribution range of *M. tridactyla* in Brazil. The aim of this study is to describe the genetic diversity within sampled populations by means of mtDNA and nuclear markers, and to evaluate the presence of genetic structure throughout the species distribution. When genetic structure was found, we searched for likely causes to explain the species' distribution along the landscape, commonly associated with either isolation by distance (IBD) or population structure driven by historical divergence. This information provides scientific resources for future management actions for the conservation of the species.

## Materials and Methods

### Sample collection

**Figure 1:**
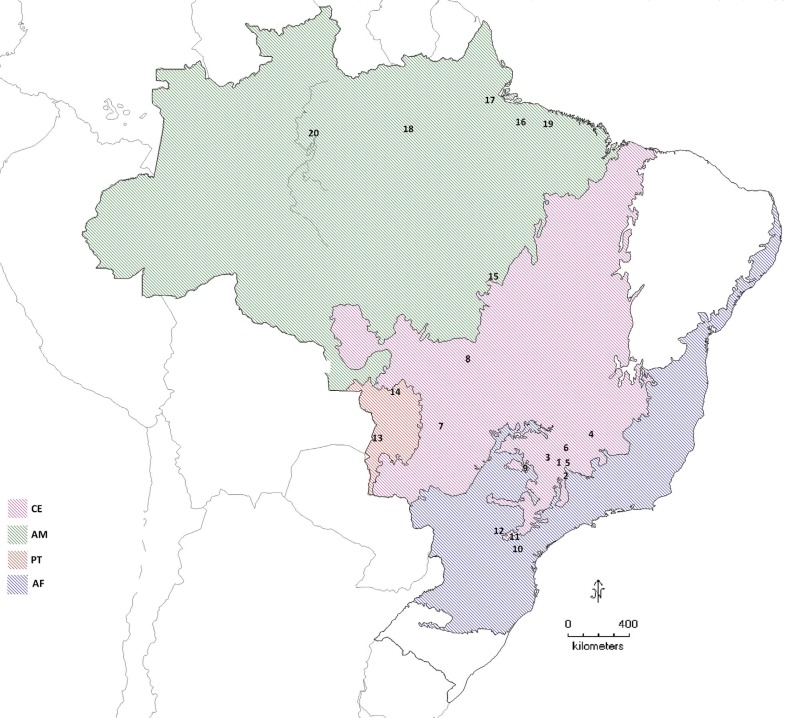
Map with localities of *M. tridactyla* individuals sampled for this study. 1- National Park of Serra da Canastra, MG; 2- Piumhi, MG; 3- Araxá, MG; 4- Dores do Indaiá, MG; 5- Doresópolis, MG; 6- Uberlândia, MG; 7- National Park of Emas, GO; 8- Nova Xavantina, MT; 9- São José do Rio Preto, SP; 10- Jaguariaíva, PR; 11- Telêmaco Borba; 12- Piraí do Sul; 13- Corumbá, MS; 14- Poconé, MT; 15- Vila Rica, PA; 16- Ilha do Marajó, PA; 17- Mazagão, AP; 18- Oriximiná, PA; 19- Belém, PA; 20- Caracaraí, RR.

Seventy-seven individuals of *M. tridactyla* were collected between 1994 and 2007 for nine Brazilian federal states (Minas Gerais, Goiás, São Paulo, Mato Grosso, Mato Grosso do Sul, Paraná, Pará, Roraima, Amapá), in 20 different localities, except for seven samples that originated from a museum collection (Museu Paraense Emílio Goeldi, MPEG), which dated from 1957-1979. Sampled tissues varied from dry skin (museum), to hair, bone and soft tissues. The original samples were collected from captive individuals of known origin, wild animals captured for ecological studies, road-killed individuals, and museum collection vouchers. All tissue samples were preserved in 95% ethanol, and stored in −20 °C. The samples covered the populations of (i) Minas Gerais state (CEMG, n=21), comprising individuals from the National Park of Serra da Canastra and other localities, (ii) Goiás state (CEGO, n=28) comprising individuals from the National Park Emas, (iii) different localities in São Paulo state (CESP, n=7) and (iv) Mato Grosso state (CEMT, n=4), all representative of the biome Cerrado (CE). Additionally, a population from the Atlantic Forest of Paraná state (AF, n=5), from the Pantanal biome (PT, n=5) of Mato Grosso and Mato Grosso do Sul states, and individuals from the Amazon Forest (AM, n=8) were also sampled. Sampling localities are displayed in Figure 1, and details regarding samples are available in Table S1 from Supplementary Material.

All biological material was collected with authorization for activity with scientific purposes emitted by SISBIO/IBAMA under the accession number 15052-1.

### Molecular Methods

Total genomic DNA was extracted from tissue samples using a standard phenol-chloroform protocol (Sambrook *et al.*, 1989). For extraction of dry skins and bone fragments a modified protocol from Holland *et al.* (2003) was used in combination with a DNA extraction Kit (DNA Tissue Kit, Qiagen). For mitochondrial DNA (mtDNA) analyses, two fragments were amplified by PCR: 450 base pairs (bp) of the first hypervariable segment from the control region (HVI), with two sets of primers, BrDi-L and BrDi-H (modified from original primers described in Arnason *et al.*, 2002 and Douzery and Randi, 1997, respectively), and Pro-L (Lara-Ruiz *et al.*, 2008) with H16498 (Ward *et al.*, 1991); and 555 bp of the Cytochrome *b* gene (*Cyt-b*) using primers CytB-L and CytB-H (Lara-Ruiz *et al.*, 2008) (or XL14733 from Kocher *et al.*, 1989, as an alternative reverse primer). For nuclear DNA (nDNA) analyses, a 700 bp fragment of the recombination activation gene (*RAG2*) was amplified with the set of primers RAG2-F220 and RAG2-R995 (Teeling *et al.*, 2001), and, finally, a 600 bp of the Y chromosome Amelogenin gene (AMELY) was amplified with primers AMELY-F2 and AMELY-R2 (Roca *et al.*, 2005), only in known male specimens.

PCR amplifications for mtDNA markers were done in 15 μL volumes containing 10X buffer, 200 μM dNTPs, 0.5 μM of each primer, and 1 unit of *Taq* DNA polymerase (Phoneutria Biotecnologia). Thermocycling conditions consisted of a denaturing step at 94 °C for 3 min, 35 cycles at 94 °C for 30 s, 50 °C for 40 s, 72 °C for 30 s, and a final extension at 72 °C for 10 s. For nDNA markers, PCR amplification was done in 10 μL volumes containing 10X buffer, 1.5 μM MgCl_2_, 200 μM dNTPs, 0.5 μM of each primer and 0.5 unit of *Platinum Taq*® polymerase (Thermo Fisher Scientific). In both cases, template DNA dilutions were used ranging between 20-100 ng/μL. Thermocycling consisted of a hotstart step at 95 °C for 9 min 45 s, 5-10 (touchdown) cycles of a denaturing step at 94 °C for 15 s, annealing at 49-54 °C for 30 s, extension at 72 °C for 80 s, and a final extension step at 72 °C for 3 min. All products were examined on a 0.8% agarose gel stained with ethidium bromide, purified with a moidified polyethyleneglycol (PEG) protocol (Santos-Júnior *et al.*, 2015), and sequenced using a MegaBACE DNA Analysis System 1000 automatic sequencer (Amersham Biosciences). All samples were sequenced at least twice, in forward and reverse directions. Museum, hair and bone samples were doubled checked.

### Data analysis

Sequence electropherograms were visually inspected using Phred v. 0.20425 (Ewing and Green, 1998), Phrap v. 0.99031 and Consed 12.0 (Gordon *et al.*, 1998), and aligned using Clustal W (Higgins and Sharp, 1988) algorithm implemented in MEGA 4.0 (Tamura *et al.*, 2007). Alignments were checked and edited by hand to account for artifacts. For autosomal data (*RAG2*), PolyPhred 5.04 (Nickerson *et al.*, 1997) was used for identifying heterozygote sites. In this case, PHASE 2.0 (Stephens *et al.*, 2001; Stephens and Donnely, 2003) was used to reconstruct haplotypes from genotypes, estimating the gametic phase. Software DNAsp 5.0 (Librado and Rozas, 2009) was used to obtain the haplotypes and their polymorphic positions for both haploid and diploid dataset.

The relationship between haplotypes and their geographical distribution was visualized through a phylogenetic network, using the median-joining (MJ) algorithm in NETWORK v. 4.6 software (Bandelt *et al.*, 1999).

Software Arlequin v. 3.5 (Excoffier and Lischer, 2010) was used to calculate haplotype (h) and nucleotide diversity (π), Θ*S* values (a measure of the population nucleotide diversity), Tajimas D, a test of selective neutrality (Tajima, 1989), population pairwise F_ST_ values and analysis of molecular variance (AMOVA) computed with pairwise differences, with 1000 permutations to test for significance at the 0.05 level. Tajimas test of selective neutrality was used to distinguish between a random (neutrality) and non-random evolving DNA sequence dataset, which may be caused by positive/balancing selection or by demographic fluctuations (expansions and contractions). When there is an excess of low frequency polymorphisms in the dataset a negative D value results, which indicates population size expansions or positive selection (Tajima, 1989). Since we only used mtDNA data for the Tajimas D analysis, which is considered mainly a neutrally evolving marker (Saccone *et al.*, 2000), we assume that a negative significant D would be most likely indicative of demographic expansion. The fixation index F_ST_ measures population differentiation based on the population frequency of genetic polymorphisms. It was estimated for each population pair, and then in groups of populations, using AMOVA, to examine the level of genetic subdivision between localities. The analyses were grouped in three different ways: (i) populations from all four biomes in distinct groups [CE][PT][AF][AM]; (ii) forested versus open vegetation biomes [AM+AF][CE+PT]; and finally (iii), using a geographic distance criterion: [CE+PT+AF] [AM].

AMOVA was also used to test for sex biased dispersion, measured in the major sampled populations (CEMG and CEGO, individuals from National Parks only), grouped by population and gender (males, M, and females, F, from each park, and in both parks). For this purpose, mtDNA (HVI+Cyt-b) and nDNA (*RAG2*) was used. Groups were tested as follows: (i) HVI+Cyt-b [CEMG+CEGO][F]/ [CEMG+CEGO][M] and (ii) RAG2 [CEMG+CEGO][F]/ [CEMG+CEGO][M].

For the mismatch distribution analysis, observed and expected pairwise differences between alleles were calculated in Arlequin (Excoffier and Lischer, 2010). It is expected to show a unimodal distribution when populations have undergone a rapid expansion, and a bimodal distribution if populations are subdivided or in demographic equilibrium (Rogers and Harpending, 1992).

In order to infer about the hypothesis of isolation by distance (IBD) we conducted the non-parametric Mantel's test, which correlates genetic and geographical distances. To access the correlation coefficient reliability, 10,000 replicates were done. The test was performed in the software Alleles in Space, AIS vs. 1.0 (Miller, 2005). To explore the existence and location of barriers to gene flow, the software Barrier vs. 2.2 (Manni *et al.*, 2004) was used. The software uses Monmoniers maximum difference algorithm (Monmonier, 1973) designed to visualize on a geographic map (represented by geographical coordinates) the trend of data constrained in a matrix, in this case, a matrix of genetic distances between all populations sampled. The triangulation edge parameters were not modified. Genetic distance matrix input was calculated with MEGA 4.0 (Tamura *et al.*, 2007). We set the initial number of barriers to four, given that we sampled four different biomes.

### Data Access

Sequence data for mtDNA and nDNA markers are publicly available at GenBank, under the accession numbers: KF543782-KF543820.

## Results

### Genetic diversity and haplotype distribution

All 77 individuals were successfully amplified and sequenced for mtDNA markers, HVI and *Cyt-b.* These fragments were analyzed jointly as one, totalizing 1005 bp. After alignment, the sequences showed 29 haplotypes distributed along four biomes (Table 1). Nuclear markers were successfully amplified and sequenced in 47 individuals for *RAG2* and in 34 individuals for AMELY. These markers were used to compare general results against mitochondrial data, once their data covered populations from CE, PT and AF, but excluded AM. *RAG2* showed eight haplotypes, and AMELY showed nine (Table 2). Sequences of haplotypes of all markers were deposited in GenBank (accession numbers KF543782-KF543820).

**Table 1:** Haplotypes for mtDNA (HVI and Cyt-b joined together), polymorphic sites and distribution per population.

Haplotype													Polymorphic Sites in mtDNA																			Haplotypes per Population					
					2	2	2	2	2	2	2	2	2	2	2	2	4	5	5	5	6	7	7	7	8	8	9	9	9	9	CE MG	CE GO	CE SP	CE MT	PT	AF	AM
	5	7	7	8	2	2	5	6	6	6	6	7	7	8	9	9	8	0	2	5	3	5	7	9	5	7	0	0	2	4							
	6	3	7	7	6	9	6	2	4	5	7	1	4	1	4	6	2	1	8	3	5	1	7	2	5	2	0	3	7	5							
H1	T	C	C	G	C	A	G	T	T	A	T	G	C	G	A	C	C	T	A	A	G	A	C	A	A	C	C	A	C	A	10	15	4	1	1		
H2	G	.	.	.	.	.	.	.	.	G	.	A	.	.	.	.	.	.	.	.	.	.	.	.	.	.	.	.	.	.	1						
H3	.	.	.		.	.	.	.	.	.	.	.	.	.	.	.	.	.	.	.	.	G	.	.	.	.	.	.	.	.	1	1					
H4	.	.	.	.	T	.	.	.	.	.	.	.	.	A	.	.	.	.	.	.	.	.	.	.	.	.	.	.	.	.	2	1					
H5	.	.	.	.	.	.	.	.	.	.	.	.	.	.	.	.	G	.	.	.	.	.	.	.	.	.	.	.	.	.	1						
H6	.	.	.	.	.	.	.	.	.	.	.	.	.	A	.	.	.	C	.	.	.	.	.	.	.	.	.	.	.	.	1	1					
H7	.	.	.	.	.	.	.	.	.	G	.	A	.	.	.	.	.	.	.	.	.	.	.	.	.	.	.	.	.	.	1	1					
H8	.	.	.	.	.	.	.	.	.	G	.	.	.	.	.	T	.	.	.	.	.	.	.	.	.	.	.	.	.	.	1						
H9	.	.	.	A	.	.	.	.	.	.	.	.	.	A	.	.	.	.	.	.	.	.	.	.	.	.	.	.	.	.				3		2	3
H10	.	.	.	A	.	.	.	.	.	.	.	.	.	A	.	.	.	.	.	.	.	.	.	.	.	.	.	.	.	.						2	1
H11	.	.	.	A	.	.	.	.	.	.	.	.	T	.	.	.	.	.	.	.	.	.	.	.	.	.	.	.	.	.	1						
H12	.	.	.	A	.	.	.	.	.	.	.	.	.	A	.	.	.	.	.	G	.	.	.	.	.	.	.	.	.	.							2
H13	.	.	.	A	.	.	.	.	.	.	C	.	.	A	.	.	.	.	.	G	.	.	.	.	.	.	.	.	.	.							1
H14	.	G	.	A	.	.	.	C	.	.	.	.	.	.	.	.	.	.	.	G	.	.	.	.	.	.	.	.	.	.							1
H15	.	.	.	.	.	.	.	.	.	G	.	A	.	A	.	.	.	.	.	.	.	.	.	.	.	.	.	.	.	.	1	1					
H16	.	.	.	.	.	.	.	.	.	G	.	A	.	A	.	.	.	.	G	.	.	.	.	.	.	.	.	.	.	.					1		
H17	.	.	.	.	.	.	.	.	.	.	.	.	.	.	.	.	.	.	.	.	.	.	.	.	.	.	.	.	.	G					1		
H18	.	.	.	A	.	.	.	.	.	.	.	.	.	.	.	.	.	.	.	.	.	.	T	G	.	.	G	.	.	.		1					
H19	.	.	.	.	.	.	.	.	.	.	.	.	.	.	G	.	.	.	.	.	.	.	.	.	.	.	.	.	.	.					1		
H20	.	.	.	.	.	.	.	.	.	.	.	.	.	.	.	.	.	.	.	.	.	.	.	G	.	.	.	.	.	.			1				
H21	.	.	.	.	.	.	.	.	.	.	.	A	.	A	.	.	.	.	.	.	.	.	.	.	.	.	.	.	.	.			1				
H22	.	.	.	.	.	.	.	.	.	G	.	A	.	A	.	.	.	.	.	.	A	.	.	.	.	.	.	.	.	.					1		
H23	.	.	.	.	.	.	.	.	.	.	.	.	T	.	.	.	.	.	.	.	.	.	.	.	.	.	.	.	.	.						1	
H24	.	.	.	.	.	.	.	.	C	.	.	.	.	.	.	.	.	.	.	.	.	.	.	.	.	.	.	.	.	.	1						
H25	.	.	.	.	.	.	.	.	.	.	.	.	.	.	.	.	.	.	.	.	.	.	.	.	C	G	.	.	.	.		1					
H26	.	.	.	.	.	.	.	.	.	.	.	.	.	.	.	.	.	.	.	.	.	.	.	.	C	.	.	G	A	.		3					
H27	.	.	.	.	.	G	.	.	.	G	.	A	.	.	.	.	.	.	.	.	.	.	.	.	.	.	.	.	.	.		1					
H28	.	.	G	.	.	.	.	.	.	.	.	.	.	.	.	.	.	.	.	.	.	.	.	.	.	.	.	.	.	.		1					
H29	.	.	.	.	.	.	A	.	.	.	.	.	.	.	.	.	.	.	.	.	.	.	.	.	.	.	.	.	.	.	1						

**Table 2:** Haplotypes for nDNA (AMELY and *RAG2*), polymorphic sites and distribution per population. *RAG2* is based in 94 sequences from 47 individuals (autosomal data).

	Haplotype	Polymorphic Sites in nDNA						Haplotype per Population						
				1	2	5		CEMG	CEGO	CESP	CEMT	PT	AF	AM
		3	7	6	1	1								
		8	1	0	0	2								
*AMELY*	A1	A	G	C	A	C		2	8		1			-
	A2	.	.	.	G	.		3		1		1		-
	A3	G	A	T	G	.						1		-
	A4	G	.	.	G	.		1						-
	A5	.	.	.	G	T				1				-
	A6	G	.	.	.	T		1	2			1		-
	A7	.	.	.	.	T		3	3	2		1		-
	A8	G	.	T	.	.							1	-
	A9	G	.	.	.	.			1					-
		2	2	3	4	7	7	CEMG	CEGO	CESP	CEMT	PT	AF	AM
		4	9	0	1	0	0							
		5	4	8	3	7	9							
*RAG2*	R1	C	C	A	A	G	C	26	28	7	1	6	2	-
	R2	.	.	.	A	.	T	2	4	2		2		-
	R3	.	.	.	A	A	.	1	4	1	1	1		-
	R4	T	.	.	A	.	.					1		-
	R5	.	G	.	A	A	.	1						-
	R6	.	G	.	A	.	.	1						-
	R7	.	.	G	A	.	.		1					-
	R8	.	.	.	G	.	.	1	1					-

**Figure 2:**
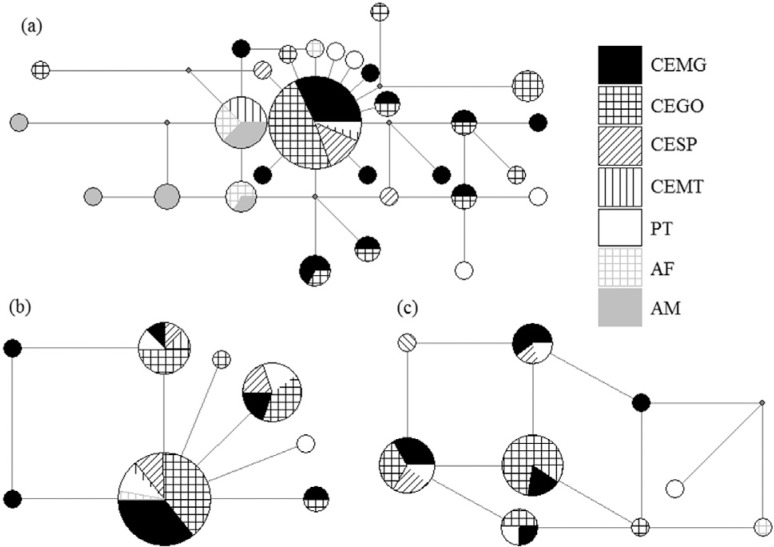
Median-joining networks of *M. tridactyla* haplotypes. (a) mtDNA, HVI and Cyt-b; (b) nDNA, *RAG2*; (c) nDNA, iAMELY. Sizes of circles are proportional to the amount of individuals carrying the haplotype. Colors and patterns are representative of populations from which individuals originated (depicted in labels).

Regarding mitochondrial data, there were 36 polymorphic sites, being 19 singletons and 17 parsimony informative sites. Total haplotype diversity was 0.8267 ± 0.0416, ranging from 0.500 in CEMT to 1.000 in PT; total nucleotide diversity was 0.002163 ± 0.001366, ranging from 0.000498 in CEMT to 0.003383 in PT (Table 3). Values of Θ*S* ranged from 0.5454 in CEMT to the highest value of 4.3685 in CEGO. In general, the results showed high haplotype diversity and moderate nucleotide diversity. In terms of populations, the highest levels of diversity were encountered in PT, despite the few individuals sampled. No particular haplotype was shared by all populations, and only two haplotypes (H1, H9) were shared among three or more populations (Table 1). Haplotype H1 was the most frequent one, found in 31 of the 77 samples, and it was found mostly in CE populations (CEMG, CEGO, CEMT and CESP), shared by only one individual in the PT population. Some haplotypes were shared between CE and PT populations, and between AF and AM populations, and only one haplotype (H9) was shared among AM, CE and AF populations. Five haplotypes were shared between CEMG and CEGO, and one between AF and AM (H10). The network of mitochondrial data exhibited a star-like shape pattern, with many haplotypes derived from the most common one (H1). Most haplotypes differ from one another by only one single mutational step (Figure 2a).

**Table 3:** Parameters of genetic diversity in each sampled population of *M. tridactyla*, and in the entire dataset. Significant Tajimas D values (p < 0.05) are marked with an asterisk (*).

Population	No. Individuals	Polymorphic Sites	Haplotype Diversity	Θ *S*	Nucleotide Diversity	Tajima's D	
						*D*	*P (Ds < Do)*
CEMG	21	12	0.7810 +/- 0.0943	3.3354	0.001838 +/- 0.001226	-1.58	0.04*
CEGO	28	17	0.7143 +/- 0.0929	4.3685	0.002022 +/- 0.001304	-1.85	0.02*
CESP	6	4	0.7143 +/- 0.1809	1.6326	0.001137 +/- 0.000952	-1.43	0.06
CEMT	4	1	0.5000 +/- 0.2652	0.5454	0.000498 +/-0.000617	-0.61	0.39
AF	5	3	0.8000 +/- 0.1640	1.4400	0.001393 +/- 0.001185	-0.17	0.49
PT	5	7	1.0000 +/- 0.1265	3.3000	0.003383 +/- 0.002425	0.08	0.57
AM	8	5	0.8571 +/- 0.1083	1.9283	0.001883 +/- 0.001363	-0.083	0.47
All	77	30	0.8267 +/- 0.0416	6.1043	0.002163 +/- 0.001366	-2.02	0.00*

Nuclear DNA data revealed the same pattern regarding haplotype distribution. The Y chromosome marker, AMELY, showed four haplotypes (A1, A2, A6, A7) shared among three or more populations, and *RAG2* showed three haplotypes (R1, R2 and R3) shared among three or more populations. Of these, all haplotypes except A1 were shared with the PT population, and only one, R1, was shared with AF (Table 2, Figure 2b, c). Since only CE, PT and AF populations could be fully analyzed for nDNA, all the following analyses regarding population expansion and population structure and biogeographic analyses will focus solely in mtDNA data.

### Population Expansion and population structure

Tajimas D for the entire sample set was −2.02 (p < 0.0001), indicating a likely demographic expansion. Only populations CEMG and CEGO also showed significant negative values of Tajimas D when analyzed separately (Table 3).

**Figure 3:**
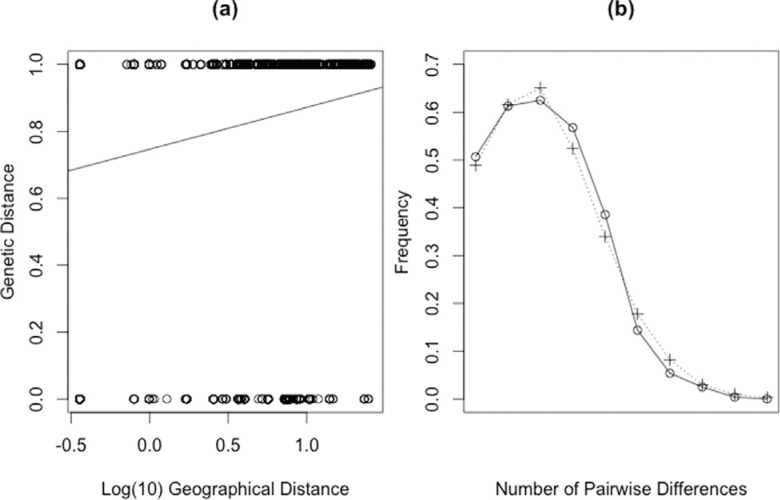
Mantel test results. (a) Plot of Mantel test showing the relationship of genetic and geographic distances (r=0.18484, p=0.9995); (b) Mismatch distribution of mtDNA data for *M. tridactyla*. Observed (Obs, dashed line) and expected (Exp, solid line) average number of pairwise differences show a unimodal fashion, compatible with a recent population expansion scenario.

The mismatch distribution graphic (Figure 3b) presented a clear unimodal fashion between observed and expected average number of pairwise differences, indicating a scenario compatible with a recent population expansion, and corroborating the star-like shape of the mtDNA network (Figure 2a).

Population pairwise F_ST_ values were low and non-significant between CE populations (CEMG, CESP and CEGO) and PT. Values were also low between populations AF and AM. Among CE populations, F_ST_ was significant only between CEMT and two other populations, CEMG and CESP. All CE populations except CEMT showed significant F_ST_ values against AM and AF (the highest CESP/AM, F_ST_=0.45252, p < 0.0001). PT showed high F_ST_ values against AF and AM as well, but was significant only against AM (Table 4).

**Table 4:** Pairwise F_ST_ values for all populations analyzed. Significant values (p < 0.05) are displayed in parenthesis and highlighted in bold.

Population (N)	CEMG	CEMT	CEGO	CESP	PT	AF	AM
CEMG (N=21)	*						
CEMT (N=4)	**0.17818** **(0.04980)**	*					
CEGO (N=28)	0.00070 (0.41113)	0.15034 (0.07227)	*				
CESP (N=7)	-0.03304 (0.83789)	**0.33211** **(0.02344)**	0.03597 (0.82424)	*			
PT (N=6)	0.03674 (0.21094)	0.24950 (0.06836)	0.08851 (0.08691)	-0.03999 (0.29980)	*		
AF (N=5)	**0.21758** **(0.01074)**	-0.02601 (0.68555)	**0.22282** **(0.00195)**	**0.32280** **(0.00195)**	0.22078 (0.08105)	*	
AM (N=8)	**0.39636** **(0.00000)**	0.18410 (0.10938)	**0.39341** **(0.00000)**	**0.45252** **(0.00098)**	**0.36288** **(0.00098)**	0.05950 (0.29688)	*

The AMOVA test revealed the highest F_ST_ value when [AM] was separated from the group [CE+PT+AF], and the lowest when groups from all four biomes were separated. The percentage of genetic variation was always higher within groups and populations than between groups (Table 5). AMOVA tests between gender (groups [CEMG+CEGO][F]/[CEMG+CEGO][M] for each marker) did not show any significant evidence of sex biased dispersal, neither in mtDNA data (HVI+Cyt-b), nor in nDNA (*RAG2*). Considering mtDNA, only 2.99% of variation could be attributed to gender, and F_ST_ was not significant (0.3607, p = 0.3704). In nDNA, similarly, 0.093% of the variation was attributed to gender groups, but was not significant F_ST_ = 0.0635, p- = 0.0861.

**Table 5:** AMOVA with groups of *M. tridactyla* populations, and percentage of variation within and between groups.

AMOVA			% Variation		
Source of Variation	F_ST_ value	P-value	Within Groups/Populations	Between Groups
[CE] [PT] [AF] [AM]	0.28169	< 0.0001	73.59	26.41
[CE+PT] [AF+AM]	0.33571	< 0.0001	69.36	30.64
[CE+PT+AF] [AM]	0.37908	< 0.0001	67.36	32.64

### Biogeographic analysis

**Figure 4:**
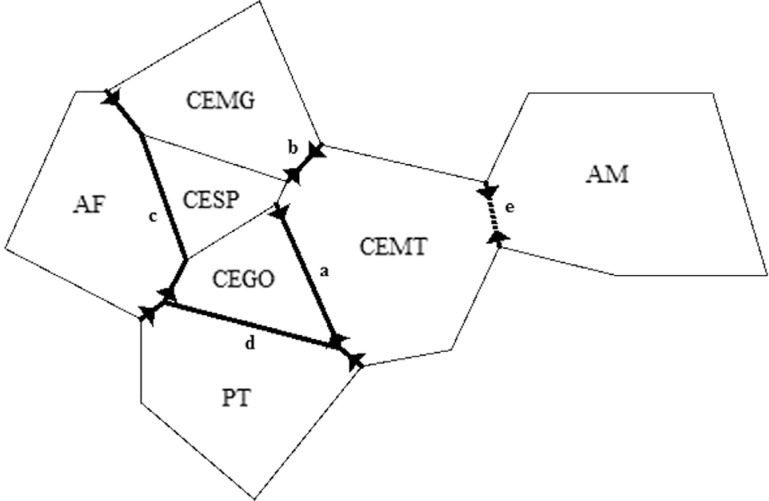
Diagram of geographic location of populations (indicated by population codes) and the placement of genetic barriers detected. Solid bold lines represent the four barriers requested to software Barrier v 2.2 (*a, b, c* and *d*), and dashed bold line represent the extra barrier requested (*e*).

The Mantel test of correlation between geographic and genetic distance was not significant (r = 0.18484, p = 0.9995) (Figure 3a). Barrier vs. 2.2 could place four different barriers between geographic regions: barrier *a* between CEGO and CEMT, barrier *b* between CEMG and CEMT, barrier *c* between three CE regions and AF and barrier *d* between PT and CEGO (Figure 4). When an additional barrier was requested in the software (five barriers), it appeared between CEMT and AM (*e*). CEMG, CEGO and CESP showed no evidence of barriers to gene flow among them.

## Discussion

This work described the general patterns of genetic diversity variation in populations of *Myrmecophaga tridactyla* along Brazilian localities. We were able to sample populations from four biomes: Cerrado (CE), Pantanal (PT), Atlantic Forest (AF) and Amazon Forest (AM), constituting the largest description of giant anteater genetic diversity available in literature to date, with mitochondrial and nuclear markers. Despite low amplification efficiency and limited sampling, the nDNA dataset corroborated largely the patterns revealed by mtDNA regarding CE, PT and AF populations.

The population from Pantanal (PT) was the genetically most diverse, followed by CEMG and CEGO, as shown by genetic parameters (haplotype and nucleotide diversity), despite the few individuals sampled. The Pantanal biome is known to be composed by different elements gathered from other biomes, such as shared fauna and flora (Prance and Schaller, 1982). This miscellaneous nature of the biome itself was likely reflected in *M. tridactyla* genetic diversity. Despite the evidence of a barrier between CE and PT (Figure 4), estimates of F_ST_ between them were low and non-significant (Table 4, Table 5), and there was a high haplotype sharing between them (Figure 2). However, since PT showed some exclusive haplotypes, wider sampling across the biome may be necessary to confirm the nature of its relationship with adjacent populations.

The population with lowest overall diversity was CEMT (for instance, an 8-fold lower Θ*S* compared to CEGO). Despite being represented by only four individuals, the region where it is located (number 8 in Figure 1) was reported to present anteater hunting activities by indigenous tribes (Leeuwenberg, 1997), and, most importantly, the region constantly suffers from severe habitat loss (Buschbacher, 2000). This population was the only one to present significant F_ST_ values against other CE populations (CEMG and CESP). This differentiation was also evidenced by the barrier analysis, where CEMT showed to be separated from CE populations, but joined with AM when four barriers were considered (the fifth barrier is displayed between them, Figure 4). Moreover, it was the only CE population to share a haplotype with AM and AF (H9). This may reflect the geographic origin of this population, placed between grassland formations (CE) and forest vegetation biomes (AM), and representing, genetically, an intermediate population.

In addition to PT, the two most diverse populations were CEMG and CEGO. Even though they were the most sampled ones, which may cause a bias, they showed the highest numbers of segregating sites and Θ*S* values (Table 3). The Θ*S* estimates are made for non-recombining DNA from the relationship between infinite-site equilibrium number of segregating sites and sample size (Watterson, 1975), and, therefore, consider the different number of samples in each population studied. Even though this parameter showed a higher value for CEMG and CEGO, corroborating their higher genetic variability. CEMG and CEGO populations are composed mostly by specimens from the National Park Serra da Canastra and National Park Emas, respectively. These are Conservation Units in Brazil, and they may represent strongholds for the species diversification, once populations are kept protected from external anthropic disturbance. The Cerrado biome, where these populations are located, has been suffering a severe and accelerated decline in its range, due mostly to the spread in agriculture borders, especially for soy bean and sugarcane production (Ratter *et al.*, 1997). The Cerrado is also considered a biodiversity hotspot for global conservation, with less than 20% of its original range left (Myers *et al.*, 2000). Indeed, these populations are highly threatened by habitat loss, and even protected areas of the Cerrado suffer from wildfires at a regular basis (either natural fires or anthropically originated ones). At times, most of the park's vegetation coverage is burned. In 1994 the entire National Park of Emas was burned, and Silveira *et al.* (1999) estimated that about 332 anteaters were killed. This factor may play a major role in the populations' diversity, once such successive bottlenecks are responsible for genetic diversity loss. Nevertheless, when Collevatti *et al.* (2007) studied the CEGO population (most individuals overlap between this study and ours) they found a high level of inbreeding and low levels of polymorphism in microsatellite loci. This outcome was also attributed to the wildfire effects. The CEMG and CEGO populations also showed significant negative values of Tajima's D, corroborating a possible scenario of repeated bottlenecks over time, followed by expansions during population recovery. Many factors place these populations in constant threats, and they should be protected for long-term maintenance of their genetic diversity. They may be suitable sources of individuals for recolonization of other populations in the vicinities, as most populations in the Cerrado (CE) showed no significant genetic distance among them.

An evident genetic differentiation was detected between CE and PT populations and AM and AF. The result of Mantel's test (non-significant correlation) suggests that IBD may be not the main factor generating genetic structuration in *M. tridactyla*. The significant F_ST_ values suggest that there is a barrier preventing some level of gene flow between these groups of populations, a hypothesis supported by barrier analysis with the Monmoniers algorithm.

At the same time, individuals from the AM population were most closely related to individuals from AF, as demonstrated in the mtDNA haplotype network (Figure 2), and by population pairwise F_ST_ values (Table 4). Even though these individuals are separated by a large distance represented mainly by the dry diagonal (Caatinga, Cerrado, Pantanal and Chaco), they both come from forest formations, which could suggest some adaptive constraints affecting population distribution. The Cerrado biome may have played a historical role as a barrier to connectivity between the two forest formations. At the same time, Cerrado vegetation also shows several fragments of deciduous and semi-deciduous forests, as well as gallery forest that constitutes a net of connections between Atlantic and Amazon biomes (Oliveira-Filho and Ratter, 1995; Vivo, 1997, Costa, 2003). Furthermore, Bigarella *et al.* (1975) suggested that both rainforests were possibly continuous in the past, and this ancient bridge can also explain the relationship between the individuals sampled from both forest biomes, as it has been reported in other studies (*e.g*. Cortés-Ortiz *et al.*, 2003; Costa, 2003; Martins *et al.*, 2007). To discuss this issue in more detail, a wider sampling covering these biomes and intermediate areas is needed.

The results presented in this study contributed to the understanding of the evolutionary history and population dynamics of the threatened giant anteater. Our data pointed out the importance of *M. tridactyla* populations of the Serra da Canastra and Emas National Parks as strongholds of diversity, an important source for future management actions for the species. Besides, it showed a marked genetic structure between Cerrado and Amazon and Atlantic forests populations, representing a historical break to gene flow, and high genetic similarity between Cerrado and Pantanal individuals. We encourage further studies with widespread populations of this species, including specimens from other biomes outside Brazil, in order to better understand its phylogeographic history and to be able to compare diversity indexes among such populations, providing useful information for conservation actions towards the species at a continental level.

## Supplementary Material

The following online material is available for this article:

Table S1 - Municipalities (or national parks) in Brazil corresponding to numbers of localities indicated in the map (Figure 1 in text). The populations to which the localities belong are also indicated
