# Supplementary material for: Population structure and genetic diversity of the giant anteater (Myrmecophaga tridactyla: Myrmecophagidae, Pilosa) in Brazil
Source: Genet Mol Biol. 2017 Feb 13;40(1):50–60. doi: 10.1590/1678-4685-GMB-2016-0104 (PMC5409771; doi:10.1590/1678-4685-GMB-2016-0104)
Supplement: Supplementary file 1 [file 1415-4757-gmb-1678-4685-GMB-2016-0104-Suppl01.pdf]

Table S1 – Municipalities (or National Parks) in Brazil corresponding to numbers of localities indicated in the map (Figure 1 in text). The populations to which the localities belong are also indicated.

Locality	Municipality or Park	Population
1	Serra da Canastra – MG	CEMG
2	Piumhi – MG	CEMG
3	Araxá – MG	CEMG
4	Dores do Indaiá – MG	CEMG
5	Doresópolis – MG	CEMG
6	Uberlândia – MG	CEMG
7	Parque das Emas – GO	CEGO
8	Nova Xavantina – MT	CEMT
9	São José do Rio Preto – SP	CESP
10	Jaguariaíva – PR	AF
11	Telêmaco Borba – PR	AF
12	Piraí do Sul – PR	AF
13	Corumbá – MS	PT
14	Poconé – MT	PT
15	Vila Rica – PA	AM
16	Ilha do Marajó – PA	AM
17	Mazagão – AP	AM
18	Oriximiná – PA	AM
19	Belém – PA	AM
20	Caracaraí – RR	AM
